# Probing Water Dissociation and Oxygen Replacement on Partially Oxygen-Covered Cu(111) by Reflection Absorption Infrared Spectroscopy

**DOI:** 10.1021/acs.jpclett.3c02004

**Published:** 2023-08-25

**Authors:** Mateusz Suchodol, Harmina Vejayan, Xueyao Zhou, Bin Jiang, Hua Guo, Rainer D. Beck

**Affiliations:** † Institute for Chemical Sciences and Engineering (ISIC), École Polytechnique Fédérale de Lausanne (EPFL), 1015 Lausanne, Switzerland; ‡ Key Laboratory of Precision and Intelligent Chemistry, Department of Chemical Physics, Key Laboratory of Surface and Interface Chemistry and Energy Catalysis of Anhui Higher Education Institutes, University of Science and Technology of China, Hefei, Anhui 230026, China; § Department of Chemistry and Chemical Biology, University of New Mexico, Albuquerque, New Mexico 87131, United States

## Abstract

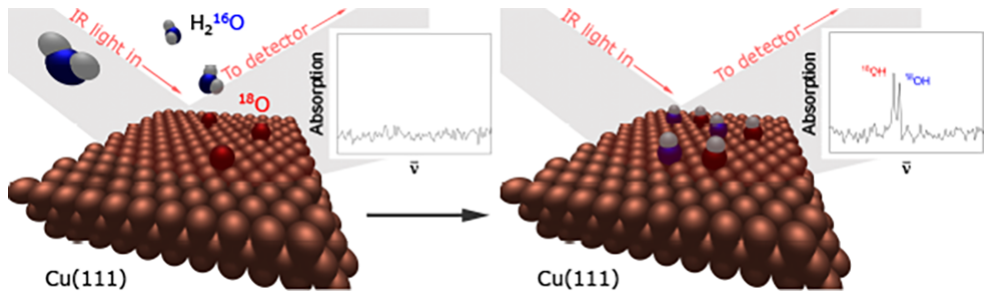

The presence of chemisorbed oxygen on the Cu(111) surface is known to strongly reduce the activation barrier for water dissociation as compared to bare Cu(111). Here, we present direct experimental evidence for the hydrogen abstraction mechanism responsible for the facile H_2_O dissociation on an O/Cu(111) surface using reflection absorption infrared spectroscopy (RAIRS) in combination with isotopically labeled reactants. We also observe that chemisorbed hydroxyl species produced by water dissociation on the O/Cu(111) surface undergo an efficient hydrogen atom transfer from trapped water molecules, leading to the rapid replacement of the initial oxygen isotope coverage and the detection of only a single hydroxyl isotopologue on the surface, in apparent contradiction with the hydrogen abstraction mechanism. In the presence of Cu_2_O oxide islands on the O/Cu(111) surface, water dissociation occurs selectively at the edges of those islands, leading to the self-assembly of isotopically ordered structures.

The promoting effect of adsorbed oxygen atoms on water dissociation has been observed for a number of transition metal surfaces. Calculations predict that the presence of O(ads) greatly facilitates water dissociation on Ag, Au, and Cu but only to a small extent on Ni(111), as summarized by Henderson.^1^ Copper surfaces are of particular interest due to the catalytic application of Cu nanoparticles in the low-temperature water-gas shift (WGS) reaction.^2−5^ Even after several decades of research due to its importance in hydrogen generation, some details governing this reaction, such as the exact nature of the catalytic sites, are still elusive. The activation barrier for water dissociation on bare Cu(110), measured from the adsorption well, was calculated by Ren and Meng to be 0.94 eV,^6^ whereas calculations by Wang and Wang yielded an activation energy of 0.28 eV for water dissociation on O/Cu(110).^7^ It has been speculated that the Cu(111) facet may be the most likely one for the reaction to happen on Cu nanoparticles,^2^ and DFT calculations suggested a high activation barrier for water dissociation on this facet of copper.^3,8,9^ The activation energy for the WGS reaction on Cu(111) facets of the Cu/ZnO/Al_2_O_3_ catalyst has been measured to be 1.17 eV by Campbell and Daube.^10^ The authors suggested an oxygen-free Cu(111) to be able to catalyze the WGS reaction, but it was also noted the oxygen influence on water dissociation could be significant.^10−13^ The presence of oxygen adatoms has been previously calculated to greatly reduce the activation barrier to 0.56^14^ or 0.32^15^ eV, measured from the water adsorption well, making the dissociation for gas-phase water essentially barrierless. With these numerous uncertainties, the rate-determining step (RDS) of the WGS reaction—water dissociation^3,11,16^—clearly still requires further investigation.

An early X-ray photoelectron spectroscopy (XPS) study of the effect of adsorbed oxygen on water dissociation on Cu(111) was published by Au et al. in 1979.^17^ When a partially oxidized O/Cu(111) surface was exposed to H_2_O at 80 K, molecular adsorption of water was detected by XPS at 533.5 eV. Upon warming the surface, a new XPS peak at 531.5 eV appeared at 173 K, which was assigned to OH(ads) at the expense of the water peak at 533.5 eV; the authors interpret their observations as a hydroxylation reaction due to dissociation of the adsorbed water on O/Cu(111). In a more recent study, Mudiyanselage et al. combined XPS and RAIRS detection to study water dissociation on Cu(111), Cu_2_O/Cu(111) and O/Cu(111).^18^ They observed the bare Cu(111) and fully oxidized Cu_2_O/Cu(111) surfaces to be inert for water dissociation. However, for a partially oxidized surface, O/Cu(111), exposed to H_2_O(g) at 90 K, they report a shift in the XPS signal from 529.5 to 531.4 eV upon annealing to 175 K, indicating that O(ads) were replaced by OH(ads) and the signal intensity increased by a factor of 2. They interpret these observations as evidence that H_2_O dissociation on O/Cu(111) proceeds by a hydrogen atom transfer mechanism according to eq 1:
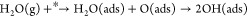
1with * corresponding to an adsorption site. Mudiyanselage et al. also used RAIRS detection to study water dissociation and report the appearance of a RAIRS absorption signal at 2690 cm^–1^, assigned to OD(ads)^18^ when an O/Cu(111) surface at 160 K was exposed to D_2_O(g). The hydrogen abstraction mechanism is supported by DFT calculations by Hao et al., with a calculated activation barrier for water dissociation via the H-transfer mechanism on O/Cu(111) to be 25.7 kJ/mol (0.27 eV) and 24 kJ/mol (0.25 eV) for 0.11 and 0.25 ML O(ads) precoverage, respectively, much lower than for the bare Cu(111) surface.^19^

In this work, we present a direct experimental observation of the hydrogen abstraction mechanism on a partially oxidized Cu(111) surface using RAIRS detection of the adsorbed hydroxyl species. Using different isotopes of oxygen (^18^O, ^16^O), we were able to observe the appearance of O–H stretch signals due to ^18^OH(ads) and ^16^OH(ads), when a partially oxidized ^18^O/Cu(111) surface was exposed to H_2_^16^O(g) at a surface temperature of 180 K. Furthermore, our isotope labeling studies revealed that the predeposited ^18^O(ads) is being replaced with ^16^O(ads) via hydrogen atom transfer between physisorbed water molecules and the hydroxyl adsorbates at *T*_s_ = 180 K, similarly to what was observed on hematite.^20^

It has been shown by Matsumoto et al.^21^ and others^22,23^ that oxidation of Cu(111) by exposure to O_2_(g) proceeds through creation and growth of Cu_2_O islands. Below, we present evidence for water dissociation occurring only at the edges of these islands, where a free adsorption site on a bare Cu atom next to an O(ads) species is available for water dissociation by the H-transfer mechanism, while the interior of the Cu_2_O islands is unreactive toward water dissociation.

The data shown in the left panel in Figure 1 are consistent with the water dissociation according to eq 2:
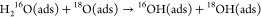
2We observed that the initial sticking coefficients of water do not change as the precoverage of oxygen on the surface is increased (Figure S1), which indicates that at *T*_s_ = 180 K the dissociation reaction occurs via a precursor-mediated mechanism, where physisorbed water molecules “roam” the Cu(111) surface and dissociate if they encounter a chemisorbed oxygen atom during their trapping time. The indirect mechanism for water dissociation on the O/Cu(111) is also consistent with the results of our DFT calculations.

**Figure 1:**
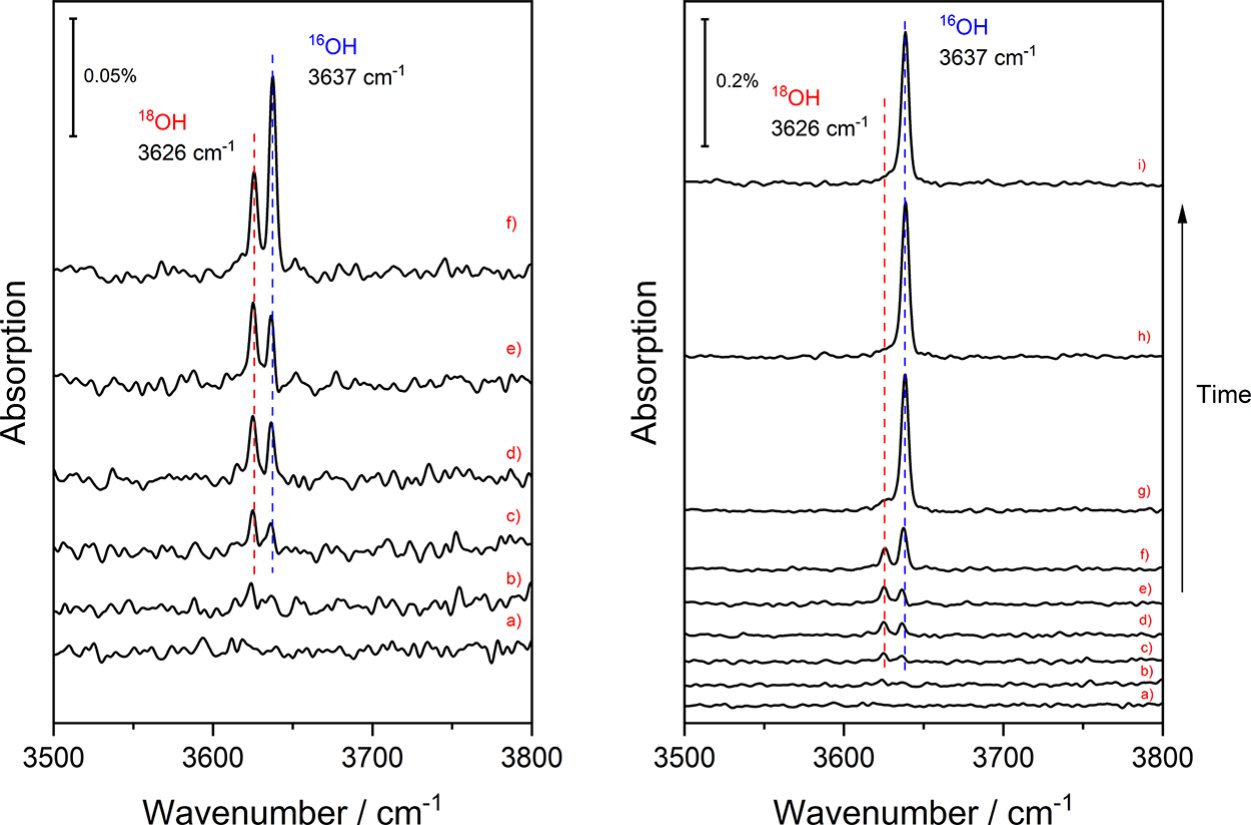
RAIRS detection of two O–H stretch absorption signals due to ^18^OH(ads) and ^16^OH(ads) resulting from H_2_^16^O(g) dissociation on a Cu(111) surface precovered with 0.07 ML of ^18^O(ads). Spectra a–e were recorded at very low H_2_O(g) partial pressure of <1 × 10^–11^ mbar; spectra f–i were recorded with increased partial pressure of H_2_^16^O(g) of 7 × 10^–10^ mbar, introduced via a leak valve. The coverage of both hydroxyl isotopologues is plotted versus time in Figure 2.

According to the H-transfer mechanism (eq 2), exposure of a partially oxidized ^18^O/Cu(111) to H_2_^16^O(g) (see Experimental Methods for details) will result in the formation of two hydroxyl species, ^18^OH(ads) and ^16^OH(ads). As shown in Figure 1 (left panel), two distinct RAIRS peaks appeared, when a partially oxidized ^18^O/Cu(111) surface was exposed to a very low partial pressure (<10^–11^ mbar, a–e) of H_2_^16^O(g) at a surface temperature of 180 K. We assign the RAIRS peaks at 3626 and 3637 cm^–1^ to the O–H stretch vibration of ^18^OH(ads) and ^16^OH(ads), respectively. Initially, both peaks are observed to increase in intensity (Figure 1, left a–e). However, when the H_2_^16^O(g) partial pressure is raised 70-fold to 7 × 10^–10^ mbar (Figure 1, right f–i), the ^18^OH(ads) peak disappears within 3 consecutive spectra (3 min), while the ^16^OH(ads) peak grows by a factor of 9. The initial growth of both peaks confirms the proposed reaction (eq 2) and the mechanism of H-abstraction, where the surface preadsorbed oxygen (^18^O) accepts a hydrogen atom from a trapped water molecule (H_2_^16^O), so that two hydroxyls are formed on the surface. We attribute the subsequent decrease and disappearance of the ^18^OH(ads) peak to a facile hydrogen exchange between the adsorbed water molecules and the hydroxyl adsorbates:
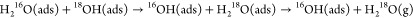
3as the rate of the decrease of ^18^OH(ads) coverage is observed to depend on the partial pressure of H_2_O(g) in the UHV chamber and, therefore, the coverage of H_2_O(ads) (Figure S5). Interestingly, isotope labeling provides an insight into this otherwise unnoticeable process. Initially, exposure of the ^18^O/Cu(111) surface to H_2_^16^O(g) causes a reaction with ^18^O(ads). The adsorbed water molecules (with an estimated trapping time of 1 μs at *T*_s_ = 180 K) interact with the hydroxyls via hydrogen-bonding-like interactions, which may lead to a hydrogen being transferred from an H_2_O(ads) molecule to an OH(ads) species. As there is only a limited amount of ^18^O(ads) and no H_2_^18^O(g) available, the surface concentration of ^18^OH(ads) decreases with time. The ^16^OH(ads) coverage continues to grow until saturation (determined by the precoverage of O(ads)) as there exists a continuous supply of H_2_^16^O(g) in the UHV chamber. This facile exchange has also been observed by us between OD (OH) adsorbates and incident H_2_O (D_2_O), resulting in the slow conversion of OD(ads) (OH(ads)) into OH(ads) (OD(ads)) in different experiments.

Our theoretical predictions for an energy barrier of about 0.17 eV for the hydrogen transfer reaction agree with those experimental observations. We conclude that, due to the limited amount of ^18^O on the surface and with continuous influx of H_2_^16^O(g), the overall reaction results in a net decrease of ^18^OH(ads) with a simultaneous build-up of ^16^OH(ads) coverage, up to the saturation level determined by the initial oxygen coverage. When we reverse the isotope labeling by using a ^16^O/Cu(111) precoverage and incident H_2_^18^O(g), the ^16^OH(ads) signal disappears and only ^18^OH(ads) can be observed. This strong dependence on the incoming flux of H_2_O could mistakenly lead researchers^24,25^ to argue against a hydrogen transfer mechanism, since for high incident H_2_O flux and a relatively slow detection technique such as RAIRS, one could fail to observe the hydroxyl peak formed due to the preadsorbed oxygen, as initially also observed in our experiments (Figure S2).

Figure 2 shows the uptake of the hydroxyl isotopologues ^18^OH(ads) and ^16^OH(ads) as a function of exposure time to H_2_^16^O(g) for an ^18^O/Cu(111) surface with an ^18^O(ads) precoverage of 0.07 ML. Each data point represents the OH(ads) peak intensity observed from a single RAIR spectrum with 60 s acquisition time (see Experimental Methods for details). For *t* < 32 min, the H_2_^16^O(g) partial pressure was in the range of 10^–11^ mbar. At this very low H_2_^16^O(g) partial pressure, the coverage of both ^16^OH(ads) and ^18^OH(ads) increases gradually with time, confirming the water dissociation mechanism by H-atom transfer from trapped H_2_^16^O(ads) to ^18^O/Cu(111). At *t* = 32 min, the partial pressure of H_2_^16^O(g) is increased 70 fold to 7 × 10^–10^ mbar, by admitting H_2_^16^O(g) into the UHV chamber through a precision leak valve. For *t* > 32 min, we observe a strong increase in ^16^OH(ads) coverage accompanied by a rapid decrease of the ^18^OH(ads) coverage due to H-atom transfer from trapped H_2_^16^O(ads) to ^18^OH(ads), according to reaction 3.

**Figure 2:**
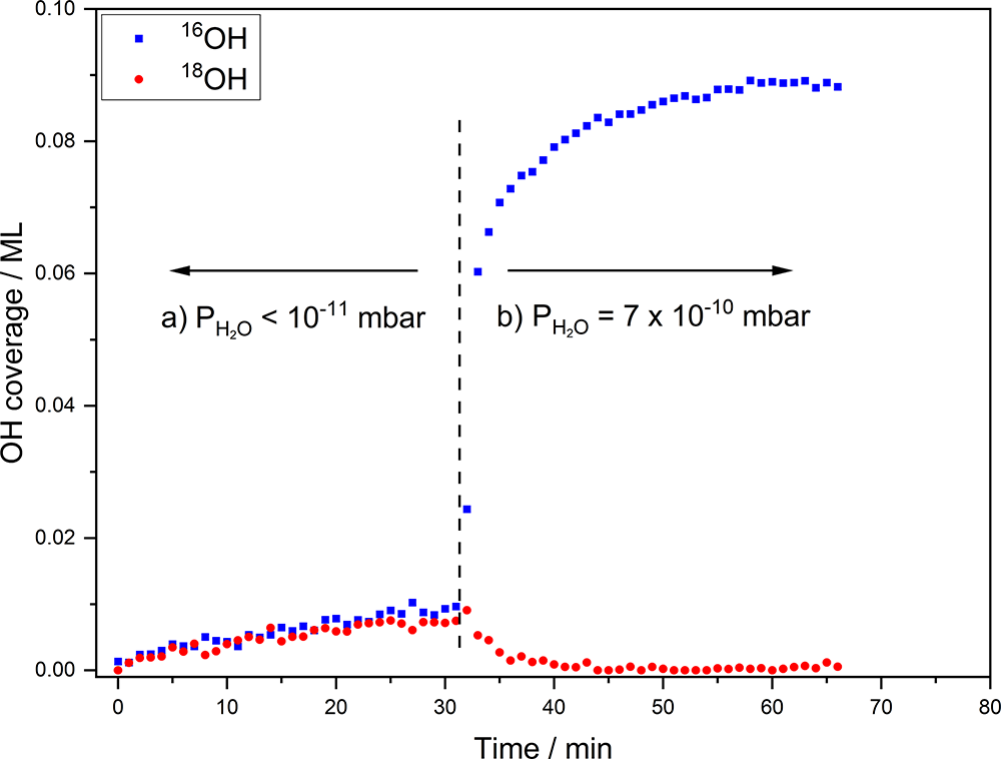
Coverage of ^16^OH(ads) and ^18^OH(ads) vs time during exposure of the ^18^O/Cu(111) surface to H_2_^16^O(g). (a) H_2_^16^O(g) partial pressure ∼10^–11^ mbar and (b) H_2_^16^O(g) partial pressure ∼70 × 10^–11^ mbar. See text for a detailed explanation.

It is well-known^21−23^ that the oxidation of Cu(111) occurs via the formation of oxygen islands, which grow by three different mechanisms, yet with the same structural composition. Matsumoto et al. highlighted those three types to be terrace, step, and added oxides.^21^ The oxygen atoms adsorbing in the vacancies of the terraces of Cu(111) lead to the growth of the terrace oxide islands. The step oxide begins growing from the step edges between two terraces. The added oxide appears as islands of copper oxide growing on top of the initially flat terrace of Cu(111), and its growth is caused by the removal of copper atoms from the vicinity of the terrace oxide. It is important to note that a fully oxidized Cu_2_O surface is not reactive toward water dissociation^18^ (confirmed by this study, data not shown), as there are no adsorption sites for the hydroxyl created from the incoming water molecules. The oxide growth initiated from the steps was determined to be stoichiometric Cu_2_O,^21−23,26^ with bare copper sites around the edges. It has been suggested by Fester et al. that oxygen-assisted water dissociation happens exactly at the edges of cobalt oxide islands.^27^ The authors studied how the OH saturation coverage depends on the radius of oxide nanoislands and observed the coverage to be inversely proportional to the radius of those islands. We note, therefore, that reaction 3 leads to the replacement of ^18^O(ads) by ^16^O(ads) selectively along the edges of the Cu_2_^18^O islands that were formed by the initial ^18^O_2_(g) dosing. If the oxygen is present in the form of Cu_2_O islands, the localized H-atom transfer reaction would lead to the formation of a self-organized isotope structure consisting of ^16^O(ads) “fences” around the Cu_2_^18^O islands. Figure 3 shows the OH(ads) saturation coverage dependence as a function of predeposited O(ads) coverage, as determined by AES. Initially, the hydroxyl saturation coverage increases linearly with the O(ads) coverage, with an approximate slope of 2, corresponding to the reaction between a water molecule and an O(ads) resulting in the creation of two OH(ads). However, the island sizes start becoming considerable at the exposure of 75L (e) of O_2_(g) to Cu(111), and the linear dependence of OH(ads) saturation coverage on the precoverage of the O(ads) breaks down. Further increases in O_2_(g) exposure (f and g) lead to decreasing OH(ads) saturation coverages, as the growth of the inside of the islands, inert to water dissociation, scales approximately with the square of their radius, *r*^2^, while the reactive edges only scale with *r*, resulting in a reduction in the amount of reactive sites available.

**Figure 3:**
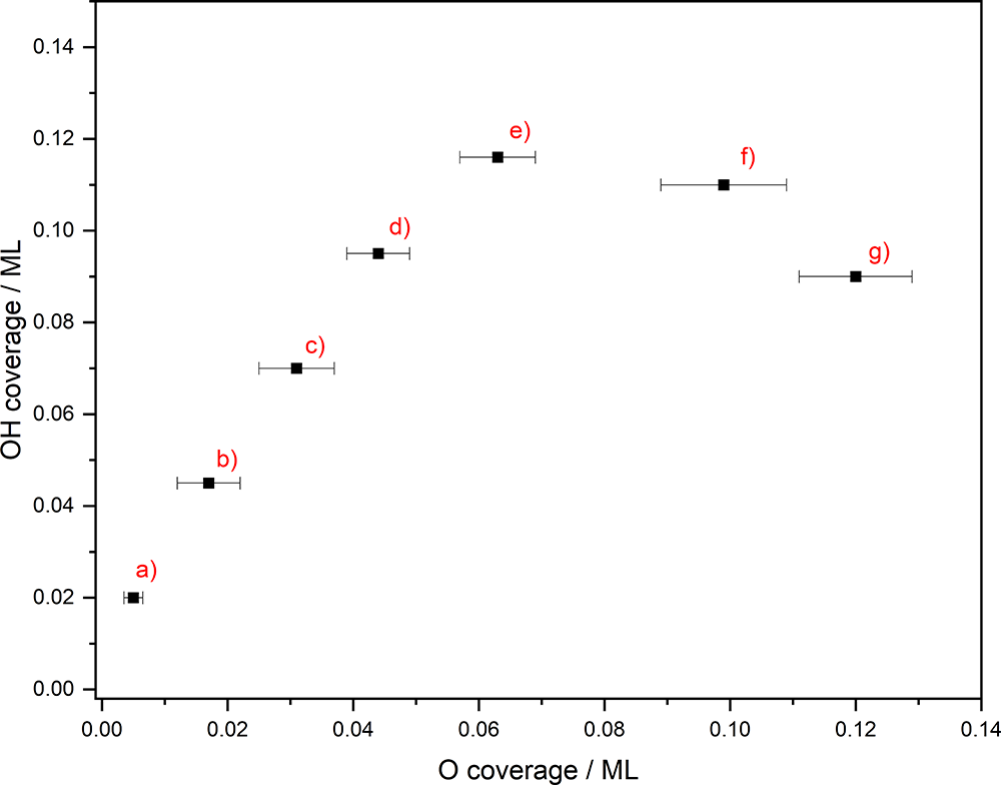
OH(ads) saturation coverage observed by RAIRS following exposure of an O/Cu(111) surface to H_2_O(g) for different initial O(ads) coverages, providing evidence for O(ads) island formation and growth with oxygen exposure. Each point corresponds to a different initial O_2_(g) exposure of the Cu(111) surface: (a) 4L, (b) 11L, (c) 23L, (d) 38L, (e) 75L, (f) 113L, and (g) 150L followed by exposure to H_2_O(g) until the OH(ads) saturation coverage is reached. As water molecules dissociate only at the island edges, the OH(ads) saturation coverage peaks near 0.06 ML O(ads) precoverage and starts to decrease with increasing island size, since an increasing fraction of the O(ads) is “hidden” on the inside of the O(ads) islands. The error bars of OH coverage (*y*-axis) are inside the data points.

Figure 4a shows the DFT calculated energies for H_2_O(g) dissociation on O/Cu(111), along with configurations of the stationary points. The calculated activation barrier for water dissociation on O/Cu(111) is 0.22 eV relative to the adsorbed water state, lower than those previously reported by Jiang and Fang (0.56 eV)^14^ and by Wang et al. (0.32 eV).^15^Figure 4a shows the reaction barrier (TS) to be 0.31 eV below the asymptote, making the direct dissociation of gas-phase water barrier-less. The transition state features a transfer of H from H_2_O to adsorbed O on the surface, leading to two adsorbed hydroxyl products. The possibility of an interaction between hydroxyls and water molecules has been studied before by Vassilev et al., albeit on a Rh(111) surface.^28^ Ab initio molecular dynamics simulations have shown hydrogen transfer to occur with an estimated proton exchange rate of 3 ps^–1^. Our calculation for the activation energy of the hydrogen transfer reaction on Cu(111), using the PBE functional, predicts a barrier of 0.17 eV (Figure 4b). Such a low barrier is consistent with rapid H-atom transfer at *T*_s_ = 180 K as the mechanism of ^18^OH(ads) disappearance, caused by hydrogen-bond-like interactions with physisorbed water molecules. Another potential pathway of OH(ads) removal would be a recombination reaction between two OH(ads), for which the activation energy has been previously calculated by Gokhale et al. to be 0.23 eV.^3^ Our own DFT calculation yields a value of 0.32 eV, as shown in Figure 4a. However, based on experimental observations described earlier, we conclude it is the hydrogen transfer reaction, not the disproportionation, that is responsible for the disappearance of ^18^OH(ads) at *T*_s_ = 180 K. It was readily seen through an Arrhenius analysis that the reaction of recombination of two hydroxyls is a thermally activated process. An Arrhenius plot yielded an activation energy of 0.42 ± 0.03 eV (Figure S3). However, we were unable to experimentally pinpoint which of the conceived steps (diffusion of OH(ads) to find a partner to react with, the recombination reaction itself, desorption of the H_2_O(ads) product) is the rate determining one; thus, this result should only be used as an estimate for the overall process. Based on computationally derived values for each of these,^3,8,15,29^ it seems plausible that desorption of H_2_O(ads), when next to an oxygen adatom, could be the elementary step with the highest activation energy.

**Figure 4:**
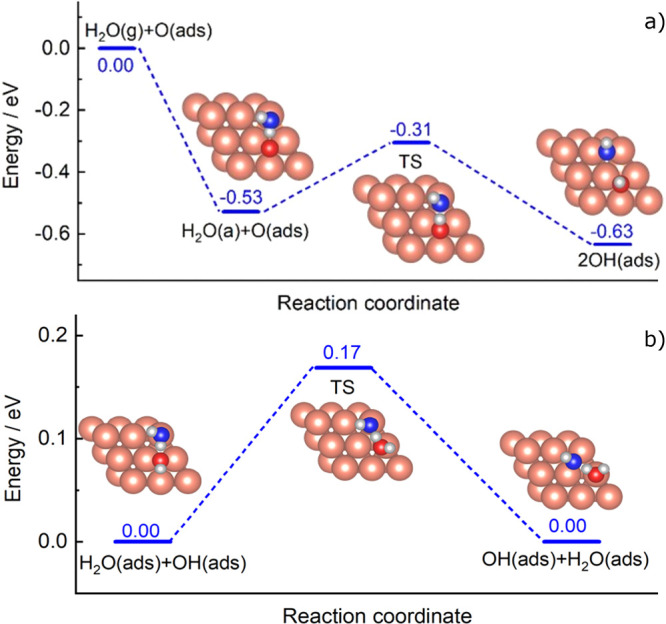
DFT-calculated structures and energetics (in eV) for (a) water dissociation reaction H_2_O(g) + O(ads) on Cu(111) and (b) H-transfer reaction: H_2_O(ads) + OH(ads) + OH(ads) + H_2_O(ads) on Cu(111).

In conclusion, we present direct experimental evidence for the hydrogen abstraction mechanism for the water dissociation reaction on a partially oxidized Cu(111) surface. Using isotopically labeled oxygen, we observed the appearance of two absorption peaks in RAIR spectra, corresponding to adsorbed hydroxyl species derived from both ^16^O and ^18^O isotopes. Isotope labeling also allowed us to probe an otherwise undetectable hydrogen atom transfer process between H_2_^16^O(ads) and ^18^OH(ads) adsorbed on the Cu(111) surface, leading to the removal of the initial oxide precoverage. These observations demonstrate the ability of the RAIRS detection to follow reactions on catalyst surfaces beyond the initial elementary step. The experimental results are supported by DFT-calculated activation energies and transition states for the H_2_O(ads) + OH(ads) and H_2_O(ads) + OH(ads) systems.

## Experimental Methods

Reflection absorption infrared spectroscopy (RAIRS) was used as a detection technique to monitor water dissociation and hydroxyl adsorbates on a Cu(111) surface at a surface temperature (*T*_s_) of 180 K. The experiments were performed in an ultrahigh vacuum (UHV) surface science apparatus of a base pressure < 2 × 10^–11^ mbar, with a Bruker Vertex V-70 FTIR spectrometer coupled to the UHV chamber. RAIR spectra were recorded with a spectral resolution of 4 cm^–1^, using a liquid-nitrogen-cooled InSb detector. Each sample spectrum is the average of 256 scans (60 s measurement time), with the background spectrum averaged over 2048 scans, recorded before oxygen deposition. Before each water exposure experiment, the Cu(111) surface was cleaned by 15 min of 1 kV Ar ion sputtering at *T*_s_ = 300 K with 2.2 μA current, followed by annealing to *T*_s_ = 900 K for 10 min. This cleaning procedure was verified by Auger electron spectroscopy (AES) to leave no detectable traces of carbon or oxygen. The clean Cu(111) surface at *T*_s_ = 300 K was then subjected to various exposures of O_2_(g), admitted into the UHV chamber via a precision leak valve, to produce partial O(ads) coverages, which were determined by AES. Following the oxygen deposition, the surface temperature was decreased to *T*_s_ = 180 K for dosing with H_2_O(g). The Cu(111) surface was exposed to H_2_O(g) either by background dosing directly into the UHV chamber (*E*_kin_ < 0.1 eV) or with a continuous molecular beam with normal incidence on the Cu(111) crystal (*E*_kin_ = 0.28 eV).

All RAIRS measurements were conducted at *T*_s_ = 180 K, which is high enough to prevent the formation of an ice layer on a bare Cu(111) surface. Temperature programmed desorption measurements (heating rate of 1 K s^–1^) showed a peak desorption rate of *T*_des_ = 164 K for molecularly adsorbed H_2_O from the Cu(111) surface (Figure S4). The hydroxyl coverage appeared to be stable at *T*_s_ = 180 K at partial pressures *P*_H_2_O_ < 1 × 10^–10^ mbar in the UHV background.

## Computational Details

DFT calculations in this work were performed with Vienna *Ab initio* Simulation Package (VASP).^30,31^ The Cu(111) surface was modeled by a four-layer slab in a 3 × 3 unit cell. The top two Cu layers and the precovered O atom were allowed to move during optimization. A vacuum space of 16 Å was added in the vertical direction to separate the slab from the periodic images. One oxygen atom was absorbed at its most stable site, i.e., the fcc site, consistent with previous DFT predictions,^32,33^ corresponding to O/Cu(111) with 1/9 ML. Generalized gradient approximation (GGA) with the Perdew–Burke–Ernzerhof (PBE)^34^ functional was used in all calculations. The ion–electron interactions were described via the projector augmented wave (PAW) method,^35^ and the kinetic energy cutoff for a plane wave basis set was 400 eV. The Monkhorst–Pack scheme was used to sample the first Brillouin zone with a 5 × 5 × 1 *k*-points mesh.^36^ The transition state (TS) for H_2_O dissociation on O/Cu(111) was determined by the dimer method^37^ and confirmed by frequency calculations.

## Supporting Information Available

Uptake curves of OH(ads) on a partially covered O/Cu(111) for different O(ads) precoverage, example spectra of high-flux experiments, Arrhenius analysis of recombinative desorption of OH(ads), TPD trace of H_2_O ice on bare Cu(111), and uptake curves of ^18^OH(ads) and ^16^OH(ads) with varied partial pressure of water (PDF)
