# Supplementary material for: Probing Water Dissociation and Oxygen Replacement on Partially Oxygen-Covered Cu(111) by Reflection Absorption Infrared Spectroscopy
Source: J Phys Chem Lett. 2023 Aug 25;14(35):7848–53. doi: 10.1021/acs.jpclett.3c02004 (PMC10494224; doi:10.1021/acs.jpclett.3c02004)
Supplement: Supplementary file 1 — jz3c02004_si_001.pdf [file jz3c02004_si_001.pdf]

Supporting Information

Probing Water Dissociation and Oxygen Replacement on Partially Oxygen Covered Cu(111) by Reflection Absorption Infrared Spectroscopy

Mateusz Suchodol[a], Harmina Vejayan[a], Xueyao Zhou[b],

Bin Jiang[b], Hua Guo[c], and Rainer D. Beck [a]*

[a] Institute for Chemical Sciences and Engineering (ISIC),

École Polytechnique Fédérale de Lausanne (EPFL), 1015 Lausanne, Switzerland

[b] Key Laboratory of Precision and Intelligent Chemistry, Department of Chemical Physics, Key Laboratory of Surface and Interface Chemistry and Energy Catalysis of Anhui Higher Education Institutes, University of Science and Technology of China, Hefei, Anhui 230026, China

[c] Department of Chemistry and Chemical Biology, University of New Mexico, Albuquerque, New Mexico, 87131, USA

***Corresponding Author:** Rainer D. Beck, rainer.beck@epfl.ch

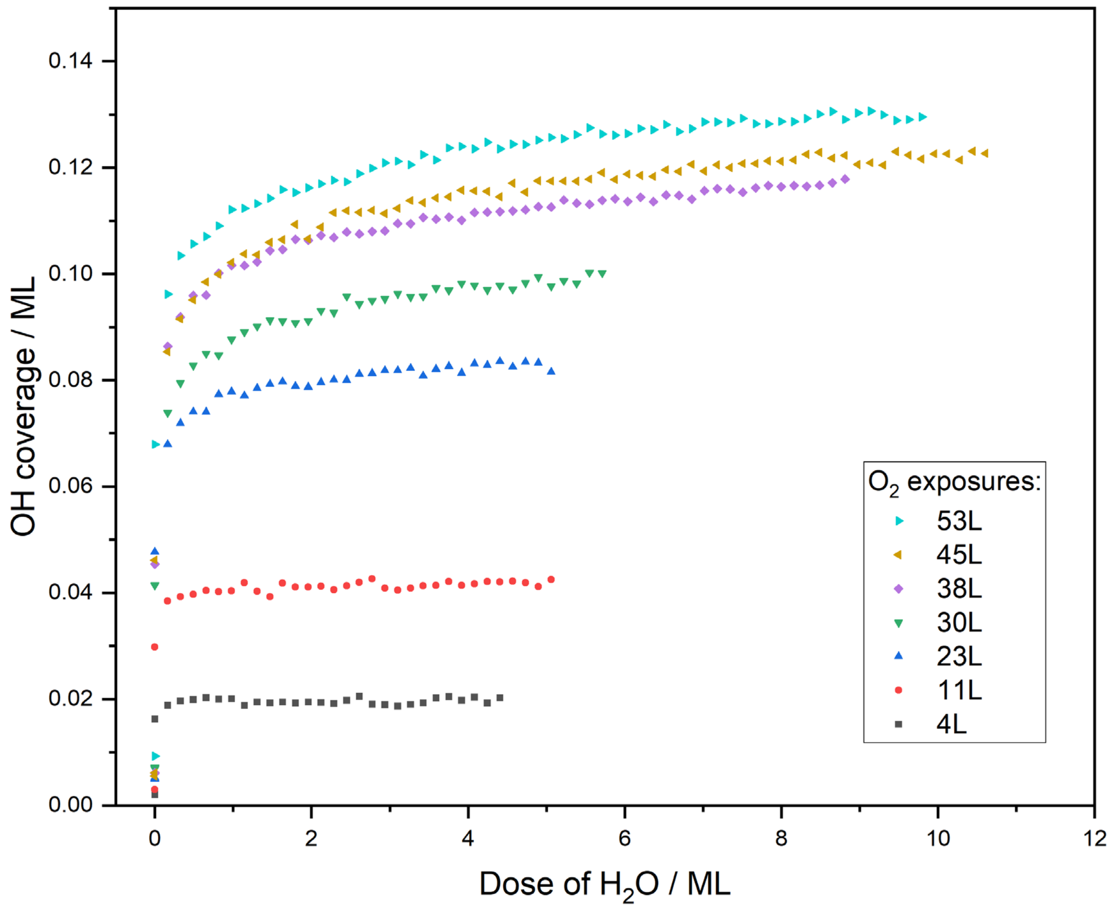

Figure S1. Uptake curves of OH(ads) on a partially covered O/Cu(111) surface. Each exposure corresponds to an approximate coverage of O(ads) of: 4L – 0.5% ML, 11L – 1.5% ML, 23L – 3% ML, 30L – 3.8% ML, 38L – 4.5% ML, 45L – 5.4% ML, 53L – 6% ML, as determined by AES. The water was background-dosed via a precision leak valve. The initial sticking coefficient for each of the curves is $S_0 = 20 \pm 5\%$, extracted as the slope of a linear fit of the first 4 data points of each uptake. No variation of S_0 with oxygen pre-coverage suggests a precursor-mediated mechanism, where a water molecule first adsorbs on the Cu(111) surface and then finds an oxygen adatom. A direct mechanism would show S_0 proportional to the initial pre-coverage of O(ads).

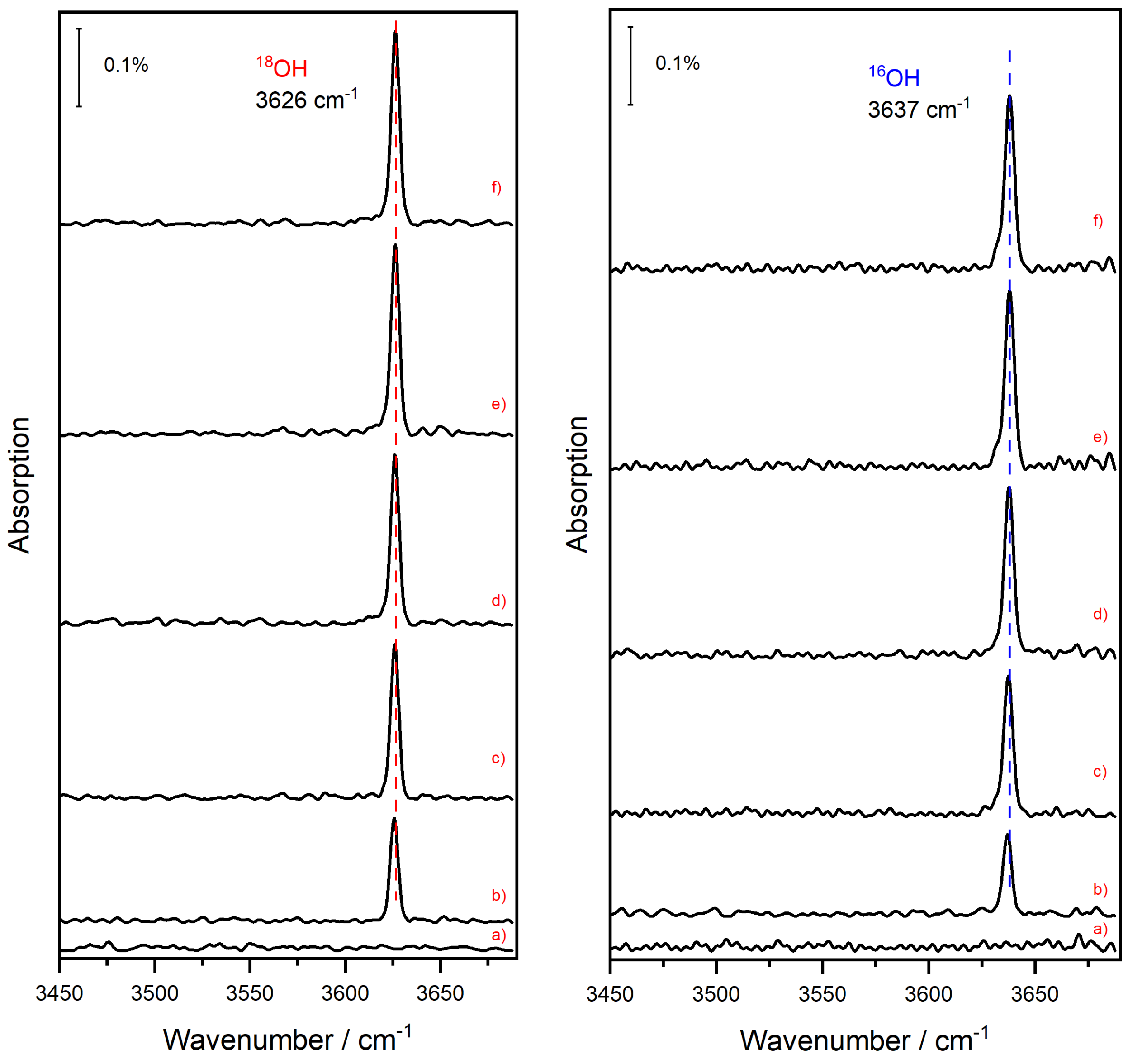

Figure S2. Initial experimental results in apparent contradiction with the H-abstraction mechanism. Left panel: a) after deposition of 0.07 ML of $^{16}O(a)$ on Cu(111), b) molecular beam (MB) exposure of 4 L $H_2{}^{18}O$ in He, c) – f) continuous MB exposure up to saturation at f). Right panel: a) after deposition of 0.07 ML of $^{18}O(a)$ on Cu(111), b) MB exposure of 4 L $H_2{}^{16}O$ in He, c) – f) continuous MB exposure up to saturation at f). Note the presence of only one hydroxyl peak, in each case corresponding to the oxygen isotope of the incoming water molecules.

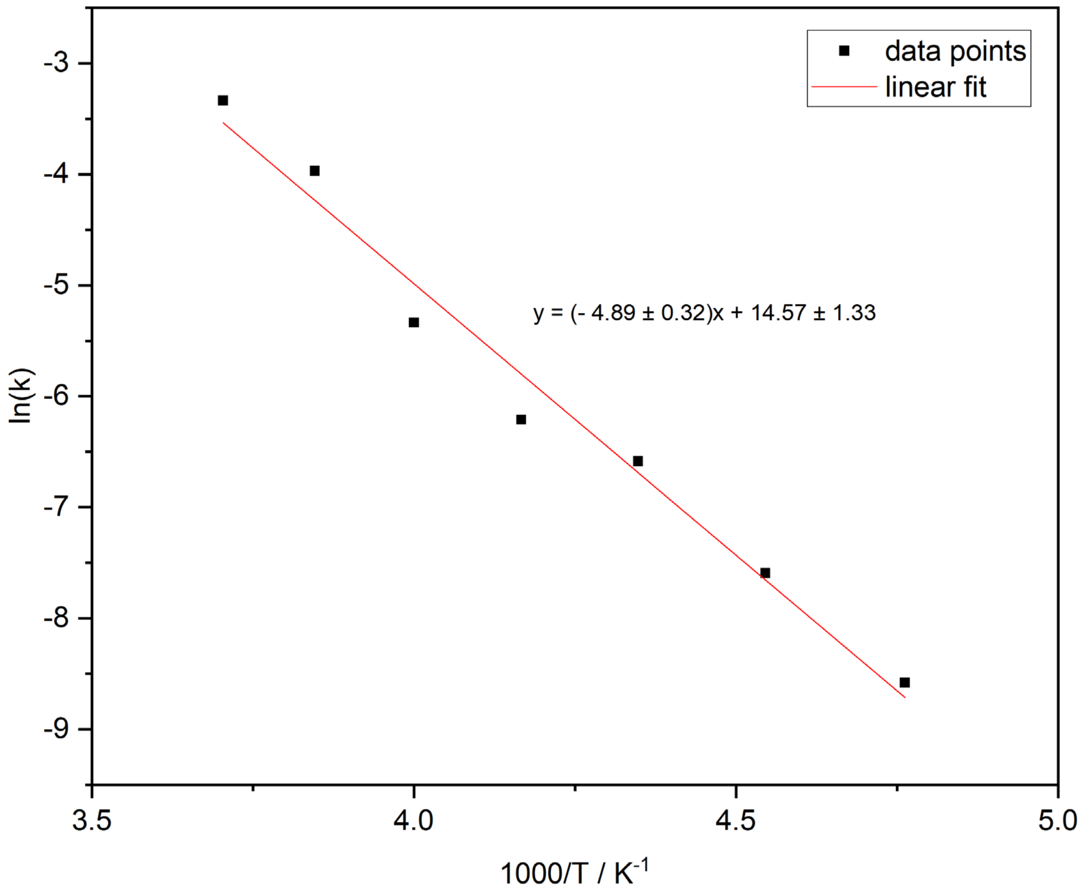

Figure S3. Arrhenius plot of recombinative desorption rate of surface hydroxyls from Cu(111). Each of the data points was extracted from a RAIRS uptake curve of OH(ads). Surface temperature was increased in steps of 10 K and kept at each new setpoint for 15 minutes. Fitting to the Arrhenius equation $ln(k) = -\frac{E_a}{R} \times \frac{1}{T} + ln\,(A)$, we find a best fit activation energy of recombinative desorption of OH(ads) of 0.42 ± 0.03 eV, with a pre-exponential factor of 10^6 s^{-1}.

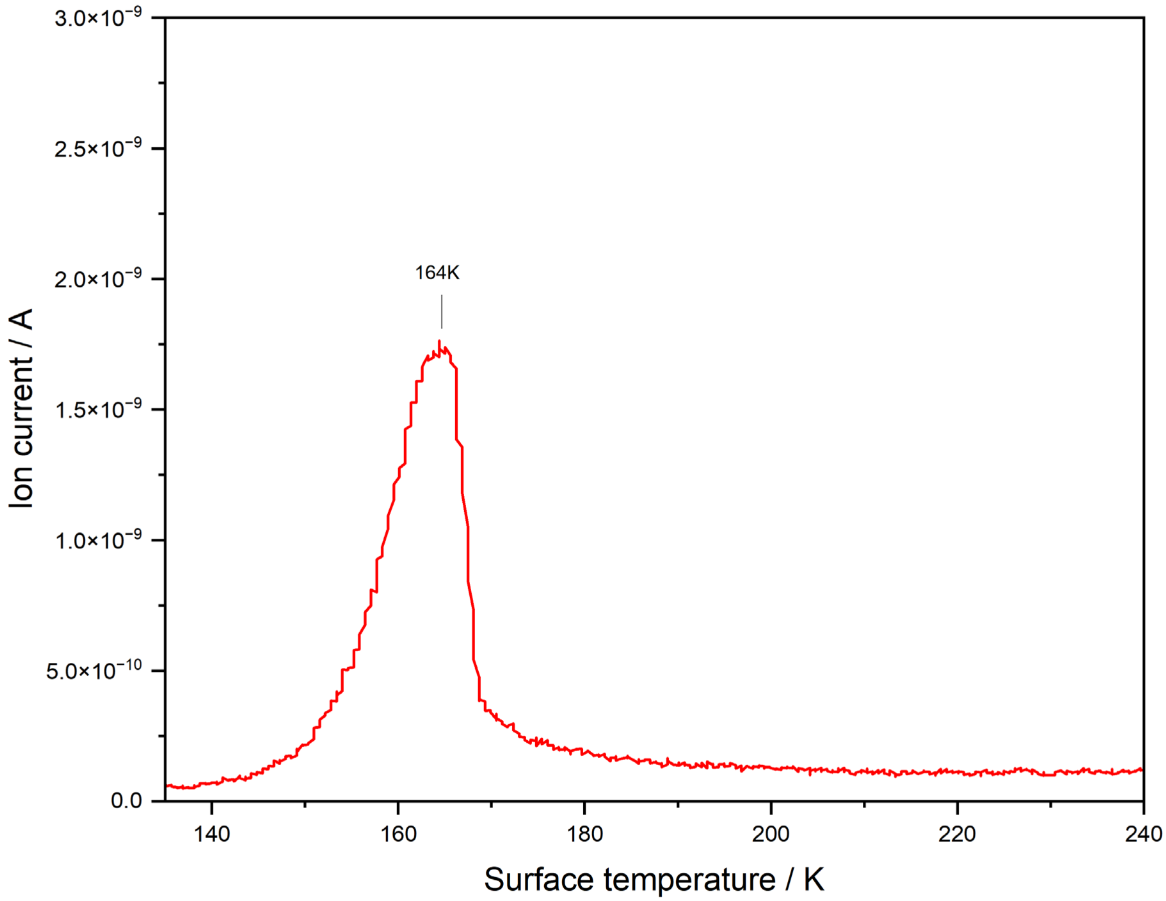

Figure S4. Temperature programmed desorption (TPD) of water (ice, heating rate 1 Ks^{-1}) from a bare Cu(111) surface after a 4.5 L exposure of H_2O at T_s = 130 K

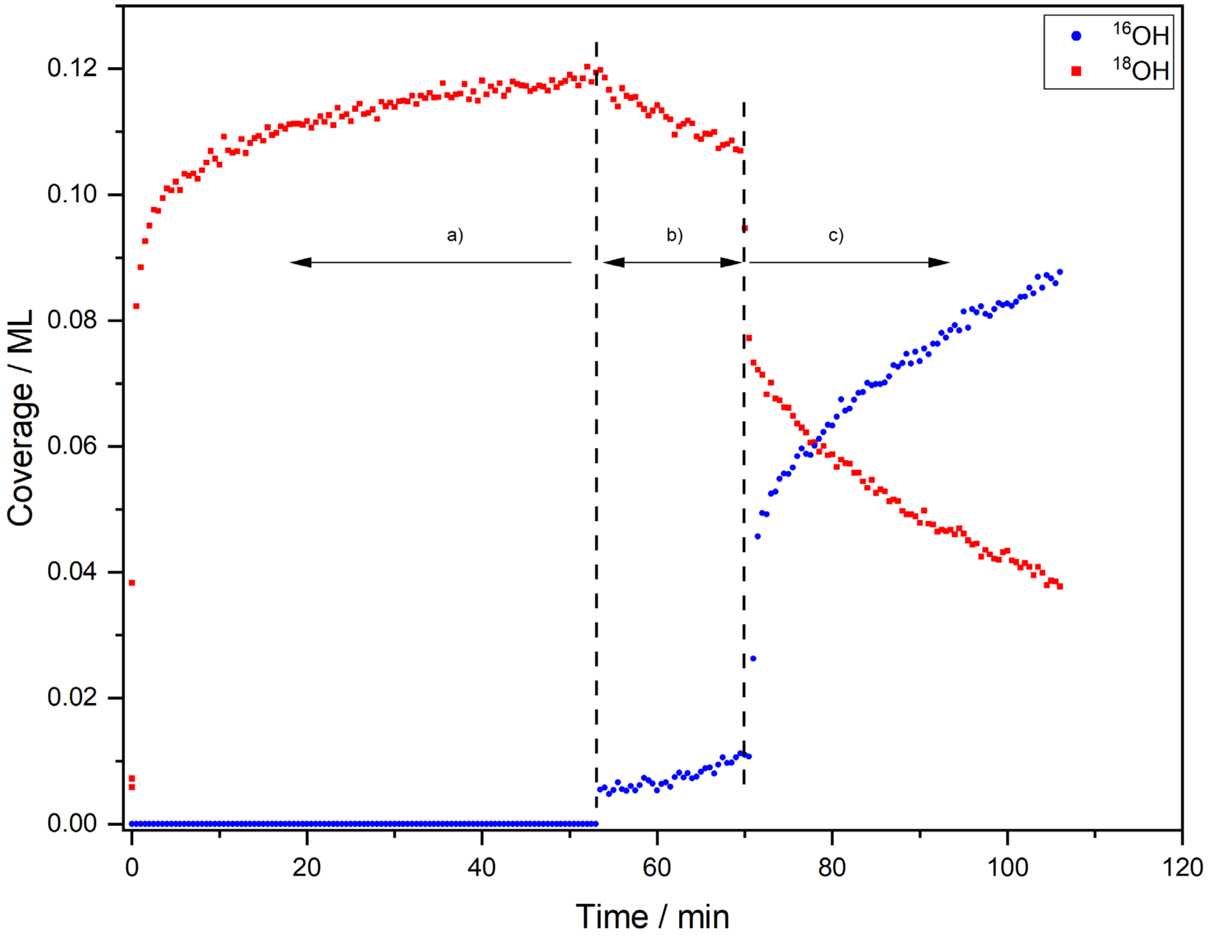

Figure S5. Coverage of both OH isotopologues plotted versus time, with 0.07 ML pre-coverage of ^{16}O(ads): a) $H_2{}^{18}O$ was introduced via a precision leak valve with a partial pressure $P = 1 \times 10^{-8}$ mbar, which resulted in only a peak of ^{18}OH(ads) growing, b) the leak valve was closed and the $H_2{}^{18}O$ pressure was continuously decreasing, allowing an absorption peak of ^{16}OH(ads) to appear at the cost of ^{18}OH(ads), c) $H_2{}^{16}O$ was introduced with a partial pressure $P = 7 \times 10^{-10}$ mbar, which accelerated the exchange of the ^{18}OH(ads) peak into ^{16}OH(ads), thus confirming the rate of this exchange depends on the partial pressure of $H_2{}^{16}O$(g).
